# pO polarography, contrast enhanced color duplex sonography (CDS), [^18^F] fluoromisonidazole and [^18^F] fluorodeoxyglucose positron emission tomography: validated methods for the evaluation of therapy-relevant tumor oxygenation or only bricks in the puzzle of tumor hypoxia?

**DOI:** 10.1186/1471-2407-7-113

**Published:** 2007-06-28

**Authors:** Bernd Gagel, Marc Piroth, Michael Pinkawa, Patrick Reinartz, Michael Zimny, Hans J Kaiser, Sven Stanzel, Branka Asadpour, Cengiz Demirel, Kurt Hamacher, Heinz H Coenen, Thomas Scholbach, Payam Maneschi, Ercole DiMartino, Michael J Eble

**Affiliations:** 1 Department of Radiotherapy, RWTH Aachen University, Germany; 2 Department of Nuclear Medicine, RWTH Aachen University, Germany; 3 Institute of Medical Statistics, RWTH Aachen University, Germany; 4 Institute of Nuclear Chemistry, Research Center Juelich, Germany; 5 Department of Pediatrics, Hospital St.Georg, Leipzig, Germany; 6 Department of Otorhinolaryngology and Plastic Head and Neck Surgery, DIAKO, Bremen, Germany

## Abstract

**Background:**

The present study was conducted to analyze the value of ([^18^F] fluoromisonidazole (FMISO) and [^18^F]-2-fluoro-2'-deoxyglucose (FDG) PET as well as color pixel density (CPD) and tumor perfusion (TP) assessed by color duplex sonography (CDS) for determination of therapeutic relevant hypoxia. As a standard for measuring tissue oxygenation in human tumors, the invasive, computerized polarographic needle electrode system (pO_2 _histography) was used for comparing the different non invasive measurements.

**Methods:**

Until now a total of 38 Patients with malignancies of the head and neck were examined. Tumor tissue pO_2 _was measured using a pO_2_-histograph. The needle electrode was placed CT-controlled in the tumor without general or local anesthesia. To assess the biological and clinical relevance of oxygenation measurement, the relative frequency of pO_2 _readings, with values ≤ 2.5, ≤ 5.0 and ≤ 10.0 mmHg, as well as mean and median pO_2 _were stated. FMISO PET consisted of one static scan of the relevant region, performed 120 min after intravenous administration. FMISO tumor to muscle ratios (FMISO_T/M_) and tumor to blood ratios (FMISO_T/B_) were calculated. FDG PET of the lymph node metastases was performed 71 ± 17 min after intravenous administration. To visualize as many vessels as possible by CDS, a contrast enhancer (Levovist^®^, Schering Corp., Germany) was administered. Color pixel density (CPD) was defined as the ratio of colored to grey pixels in a region of interest. From CDS signals two parameters were extracted: color hue – defining velocity (v) and color area – defining perfused area (A). Signal intensity as a measure of tissue perfusion (TP) was quantified as follows: TP = v_mean _× A_mean_.

**Results:**

In order to investigate the degree of linear association, we calculated the Pearson correlation coefficient. Slight (|r| > 0.4) to moderate (|r| > 0.6) correlation was found between the parameters of pO_2 _polarography (pO_2 _readings with values ≤ 2.5, ≤ 5.0 and ≤ 10.0 mmHg, as well as median pO_2_), CPD and FMISO_T/M_. Only a slight correlation between TP and the fraction of pO_2 _values ≤ 10.0 mmHg, median and mean pO_2 _could be detected. After exclusion of four outliers the absolute values of the Pearson correlation coefficients increased clearly. There was no relevant association between mean or maximum FDG uptake and the different polarographic- as well as the CDS parameters.

**Conclusion:**

CDS and FMISO PET represent different approaches for estimation of therapy relevant tumor hypoxia. Each of these approaches is methodologically limited, making evaluation of clinical potential in prospective studies necessary.

## Background

Tumor hypoxia has been associated with malignant progression, representing an increasing probability of recurrence, loco regional spread and distant metastases. In addition, the hypoxic fraction in solid tumors reduces their sensitivity to conventional treatment modalities, modulating therapeutic response to ionizing radiation or certain chemotherapeutic agents [5,8,10,25]. Consequently detection and monitoring of tissue oxygenation can be important for modifying therapeutic strategies, including local dose escalation for radiotherapy or selection of chemotherapeutic agents with better impact even in hypoxic tumors. The causes of tumor hypoxia are complex including multiple factors determining tumor blood flow such as perfusion pressure, viscous and geometric resistance to flow, arteriovenous shunts and interstitial fluid flow.

Numerous different approaches have been made to identify hypoxia in tumors [12,13]. The computerized polarographic needle electrode system (pO_2 _histography) represents one standard for measuring tissue oxygenation in human malignancies [28]. However, it is an invasive method being confined to superficial, well accessible tumors or lymph node metastases.

A non-invasive approach to detect hypoxia in tumors is the positron emission tomography (PET) with nitroimidazole derivates. Nitroimidazoles are recognized to bind selectively to hypoxic cells [23] and are reduced intracellular. In hypoxic cells re-oxidation is hindered, leading to intracellular accumulation of nitroimidazole. Radiolabelled nitroimidazole used in positron emission tomography is therefore highly suitable to determine tumor tissue oxygenation.

We present the results of our analyses of a cohort of patients with head and neck malignancies, being examined with direct, invasive measurements of tissue oxygenation by pO_2 _polarography and non-invasive [^18^F] Fluoromisonidazole (FMISO) and [^18^F] Fluorodeoxyglucose (FDG) positron-emission-tomography (PET) for determination of tumor oxygenation. In addition two factors related to oxygen delivery, the tumor vascularisation and tumor perfusion were measured by contrast-enhanced color duplex sonography (CDS).

## Methods

### Patients

Lymph node metastases of 38 patients with histologically verified head and neck malignancies (36 patients with squamous cell cancer, one patient with lympho-epithelial cancer and one patient with Hodgkin's lymphoma) were enrolled in a prospective clinical evaluation between October 2002 and January 2005. Standard sonographic, CT and PET criteria were used for the diagnosis of metastatic lymph nodes. In 20 patients the diagnosis were confirmed histologically. Patients underwent the following measurements within one week: contrast-enhanced color duplex sonography (CDS), FDGPET, FMISOPET and polarographic pO_2 _measurement (last measurement). None of the patients had previously been treated for their malignancies. Because of different treatment modalities (surgery followed by radio- or radio-/chemotherapy, primary radio-/chemotherapy and chemotherapy alone) no clinical analysis was performed. The study was approved by the medical ethical committee of the University Aachen, Germany. After explanation of the rationale, risks, and benefits of the examination, informed consent was obtained from all patients.

Measurements were performed in one lymph node metastases in each patient. In order to ensure measurements in the same suspected lymph nodes, sonographically examined lymph nodes were marked on diagnostic CT scans or in the case of lymph node conglomerates, the extension of the scanned node was marked on skin. PET examinations were realized in most of the patients within two days (maximum time interval four days) using skin markers and positioning lasers for reproducible data acquisition resulting in a corresponding slice location. No immobilization device was used. Sonographic studies, selection of representative images and parameter calculation as well as polarographic measurement were performed by one person each respectively, resulting in an objective and independent data acquisition. As detailed information of all used techniques was published by our group [3,6,27,34] only a description of the essential aspects of the different measurement procedures is given.

### Color Duplex Sonograpy (CDS)

Technical details and adjustment of the sonography device were used as previously published [3,6,27]. In order to visualize as many vessels as possible a contrast enhancer (Levovist, Schering Corp., Berlin, Germany) was administered. We used a solution of 4 g Levovist^® ^in 11 ml sterile water for injection. 5.5 ml were given as a bolus whereas 5.5 ml were infused with 300 ml/h. CDS was performed in 32 patients.

### Color pixel density (CPD)

This examination could be realized in 32 patients. All sonographic studies were recorded on digital video. From these video recordings, representative horizontal and longitudinal scans were selected for assessment. Depending on the size of the investigated nodes, this resulted in 5 to 16 (mean = 8.13;standard deviation (SD) = 4.38) images of each lymph node. For visualization of tumor vascularisation, the maximal systolic phases were used. Sonographic studies, selection of representative images and parameter calculation were performed by one person each, respectively. In a region of interest, representing the extent of vascularisation in the investigated slice, CPD was defined as ratio of colored pixels to gray pixels. The mean CPD was calculated in order to find a representative value for imaged vascularisation.

### Quantification of perfusion

In the course of the study the commercially software (PixelFlux^®^, Chameleon-Software Corp., Leipzig, Germany) was available. In 18 patients this software was used for evaluation of blood flow dynamics in the lymph node metastases until now. Cervical lymph nodes were examined at the largest diameter to record perfusion signals of intranodal vessels. All sonographic studies were recorded and perfusion signals of every single image were read out automatically from the region of interest (ROI). The ROI was defined in advance, encompassing the whole node's area, sparing out surrounding tissue.

The new technique allows a simultaneous measurement of the classical resistance index (RI) and pulsatility index (PI) in all vessels of the region of interest (ROI) [27]. Every single pixel in each vessel is traced through a complete heart cycle. Changes of color hue – representing flow velocity at this point – are measured and RI as well as PI is calculated. By this approach tissue-RI (TRI) and tissue-PI (TPI) for each point of a complete heart cycle were calculated, representing mean PI and RI of all vessels inside the ROI. Signal intensity as a measure of tissue perfusion (TP) was quantified as follows: TP = v_mean _× A_mean _with A = part of the ROI filled with color signals and v = velocity values of all pixels inside the ROI changing due to heart action.

### Positron-Emission-Tomography (PET)

In 24 patients FDG PET as well as FMISO PET examinations could be performed. All PET studies were carried out using an ECAT EXACT 922/47^® ^scanner with an axial field of view of 16.2 cm (Siemens CTI, Knoxville, TN, USA). The spatial resolution in the transaxial and axial orientation of this PET is nearly isotopic. In the reconstructed images, the full width at half maximum (FWHM) is about 6.0 mm measured at the center of the field of view (FOV) using a ramp filter with a 0.5 cut-off frequency.

FDG PET of the tumor region was performed 71 ± 17 min after intravenous administration of 264 ± 46 MBq FDG, applying the whole-body tool with 8 min emission scanning and 4 min transmission scanning for each bed position. After correction for attenuation using the transmission scan optimized by a segment μ-map with empirical attenuation coefficients, the data were reconstructed with the OSEM algorithm [14]. All patients fasted for at least 6 h before examination, verified by determining blood glucose level (mean = 90.8 mg/dl; SD = 16.0 mg/dl). None of the patients showed a higher concentration than 120 mg/dl, so treatment with insulin prior to examination was not necessary.

FMISO PET consisted of one static scan of the relevant region – as defined by sonography or computed tomography – performed 120 min after intravenous administration of 314 ± 42 MBq FMISO. The acquisition time included 15 min emission scanning followed by 4 min transmission scanning. Attenuation correction and reconstruction processing was done according to FDG PET. Three venous blood samples (after 120-, 125- and 130 minutes) were taken at each static scan. After correction according to the half-life period the mean values were calculated. The radioactivity concentration was measured in a calibrated well counter (Spectrum Master 92X, EG&G Ortec, Oak Ridge, TN). After correction for decay the mean values were calculated.

The tumor was defined according to the image data of the FDG-PET and the puncture computer tomography (CT) scans. In cases where the tumour was not clearly visible in the FMISO scan, FDG data were used to delineate the malignant lesion and define a region of interest. Rectangular regions of interest depending to the tumor size were placed over the tumor and ipsilateral nuchal muscles in order to calculate FMISO tumor to muscle ratios (FMISO_T/M_). To calculate tumor to blood ratios (FMISO_T/B_) at 120 min after administration of FMISO, the average radioactivity concentration of the three blood samples was used.

For FDG PET, mean and maximum standardized uptake values (SUV) of the tumor were calculated after normalization of the radioactivity concentration to the injected radioactivity and the body weight. Additionally, the mean SUV was approximately corrected for partial volume effects by applying recovery coefficients obtained from phantom studies [14]...

### Polarographic pO2 measurement

Tumor oxygenation was measured in 36 patients with polarographic needle electrodes, using a pO_2_-histograph (Eppendorf, Hamburg, Germany). Sterile polarographic needle electrodes with stainless steel shafts and a mean diameter of 300 μm were used. For each patient the needle electrodes were placed and guided CT-controlled in the tumor after visual matching of CT- and PET-scans without general or local anesthesia, avoiding larger necrotic areas by preceding diagnostic CT scans. In this way we realized 95–400 single pO2 measurements per lymph node (mean = 225; SD = 61) resulting in a representative distribution of pO2 values. The relative frequency of pO_2 _readings with values ≤ 2.5 mmHg, ≤ 5.0 mmHg and ≤ 10.0 mmHg as well as mean and median pO_2 _were calculated to asses the biological and clinical relevance of oxygenation measurement [30].

### Statistical methods

Data are summarized by calculating relative frequencies, as well as suitable measurements of location and variation. The degree of linear relationship between different, polarographically measured parameters of tumor hypoxia, CPD, TP and the FDG-, and FMISO-PET parameters investigated by computing Pearson correlation coefficients and displayed graphically using scatter plots.

All statistical analyses were performed using the SPSS^®^12.0 statistical analysis software package.

## Results

### Color Duplex Sonography

The interindividual range of mean color pixel density in the investigated lymph nodes was between 2.4% and 16.4% (mean = 8.1%; SD = 2.4%). The average TP was 0.065 cm/s with a range from 0.012 cm/s to 0.173 cm/s and a SD of 0.051 cm/s. A mean number of 8.0 (range: 5–16; SD = 4.4) recorded slices per measurement were evaluated.

### Positron-Emission-Tomography

Mean and maximum SUV of FMISO (average FMISO_SUVmean _= 1.69, SD = 0.40; average FMISO_SUVmax _= 1.98, SD = 0.50) and FDG (average FDG_SUVmean _= 8.13, SD = 3.45; average FDG_SUVmax _= 9.79, SD = 4.41) as well as tumor-to-muscle ratios of FMISO (averageFMISO_T/M _= 1.57, SD = 0.34) and tumor-to-blood ratios of FMISO (average FMISO_T/B _= 1.13, SD = 0.25) were calculated for the total tumor regions.

### Polarographic pO2 measurement

The average median pO_2 _was 12.5 mmHg (range: 0.1–41.1 mmHg; SD = 10.3 mmHg). The average mean pO_2 _was 17.6 mmHg (range: 8.8–36.0 mmHg; SD = 7.3 mmHg). The mean proportion of pO_2 _values ≤ 2.5 mmHg was 29.3% (range: 0.0–58.5%; SD = 18.4%), of values ≤ 5.0 mmHg 38.4% (range: 7.0–73.6%; SD = 18.1%) and of values ≤ 10.0 mmHg 48.9% (range: 13.0–78.7%; 18.2%).

### Correlations

**Figure 1:**
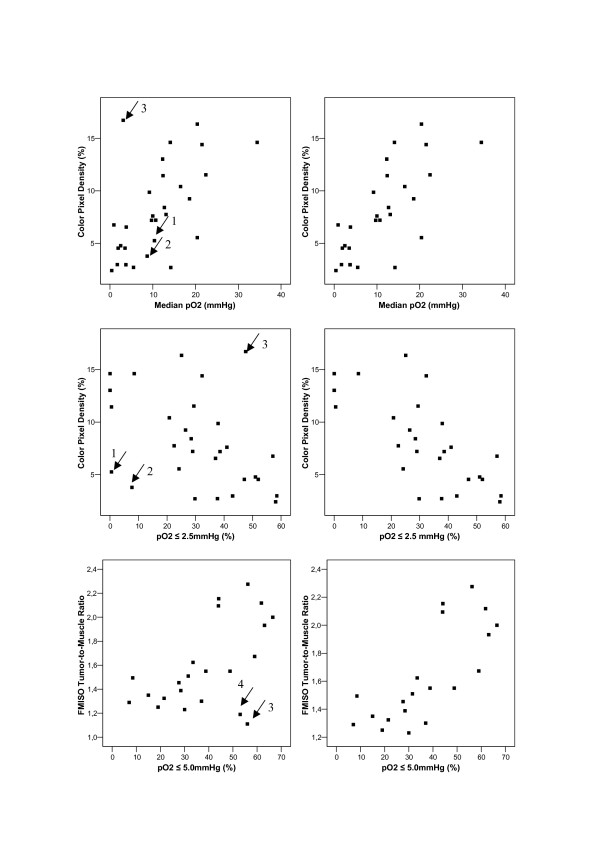
Scatter plots visualizing type of association between selected polarographic parameters (median pO_2_, hypoxic fraction ≤ 2.5 mmHg and ≤ 5.0 mmHg) and color pixel density (CPD) as well as FMISO tumor to muscle ratio (FMISO_T/M_) after 2 h (→ marked outliers). Left graphs: scatter plots using all available data values. Right graphs: corresponding scatter plots after exclusion of marked outliers identified in the left graph.

In order to detect possible relations between the different relevant polarographically measured parameters of tumor hypoxia, FMISO_T/M_, FMISO_T/B_, CPD and TP, we calculated the Pearson correlation coefficient. Correlations are listed in Table 1. To emphasize relevant correlations (r>0.4) bold numbers were used. Only a slight association between FMISO_T/B _and the hypoxic fraction ≤ 2.5 mmHg could be detected. There was no relevant correlation between FMISO_T/B _and the other polarographically, as well as sonographically, measured parameters. There was also no relevant association between TP and the hypoxic fractions ≤ 2.5 mmHg as well as ≤ 5.0 mmHg. When graphically analyzing the observed correlations through an evaluation of the corresponding scatter plots (Figure 1), there were two patients (patient1; patient2) with obviously low color pixel density, and mean/median pO_2 _values based on inhomogeneous distribution of the different hypoxic fractions with a high percentage of readings ≤ 10 mmHg and low percentage of readings ≤ 2.5 or ≤ 5.0 mmHg.

**Table 1:** Correlations between different relevant polarographically measured parameters of tumor hypoxia, FMISO PET, CDS and FDG PET data based oxygenation values

	≤2.5 mmHg	≤5.0 mmHg	≤10.0 mmHg	Median pO_2_	Mean pO_2_	FMISO_T/M_	FMISO_T/B_	CPD	TP	FDG_SUVmax_	FDG_SUVmean_
≤2.5 mmHg						**r = 0.746**	**r = 0.476**	**r =-0.735**	*r = -0.355*	*r = 0.302*	*r = 0.276*
N	36					20	20	28	*16*	*20*	*20*
≤5.0 mmHg	**r = 0.934**					**r = 0.757**	**r = 0.478**	**r =-0.786**	**r =-0.527**	*r = 0.161*	*r = 0.123*
N	36	36				20	20	28	16	*20*	*20*
≤10.0 mmHg	**r = 0.765**	**r = 0.903**				**r = 0.703**	**r = 0.435**	**r =-0.788**	**r =-0.604**	*r = 0.021*	*r = -0.013*
N	36	36	36			20	20	28	16	*20*	*20*
Mean pO_2_	**r =-0.610**	**r =-0.768**	**r =-0.902**			**r =-0.589**	*r = -0.278*	**r = 0.720**	**r = 0.607**	*r = 0.167*	*r = 0.174*
N	**36**	36	36	36		20	*20*	28	16	*20*	*20*
Mean pO_2_	**r =-0.485**	**r =-0.676**	**r =-0.837**	**r = 0.923**		**r =-0.582**	*r = -0.272*	**r = 0.576**	**r = 0.508**	*r = 0.166*	*r = 0.161*
N	36	36	36	36	36	20	*20*	28	16	*20*	*20*
FMISO_T/M_	**r = 0.558**	**r = 0.551**	**r = 0.462**	**r =-0.413**	*r = -0.376*						
N	22	22	22	22	*22*	24					
FMISO_T/B_	**r = 0.401**	*r = 0.396*	*r = 0.342*	*r = -0.215*	*r = -0.200*	**0.557**					
N	22	*22*	*22*	*22*	*22*	24	24				
CPD	**r =-0.475**	**r =-0.537**	**r =-0.662**	**r = 0.590**	**r = 0.468**	**r =-0.503**	*r = -0.270*				
N	31	31	31	31	31	18	*18*	32			
TP	*r = -0.145*	*r = -0.303*	**r =-0.592**	**r = 0.604**	**r = 0.519**	**r =-0.509**	*r = 0.004*	**r = 0.784**			
N	*18*	*18*	18	18	18	9	*9*	18	18		
FDG_SUVmax_	*r = 0.241*	*r = 0.101*	*r = -0.056*	*R = 0.205*	*r = 0.219*	*r = 0.159*	**r = 0.403**	*r = 0.105*	*r = 0.179*		
N	*22*	*22*	*22*	*22*	*22*	*24*	24	*18*	*9*	24	
FDG_SUVmean_	*r = 0.238*	*r = 0.087*	*r = -0.062*	*R = 0.193*	*r = 0.193*	*r = 0.108*	*r = 0.329*	*r = -0.077*	*r = 0.103*	**r = 0.982**	
N	*22*	*22*	*22*	*22*	*22*	*24*	*24*	*18*	*9*	24	24

Correlations after exclusion of outliers in the marked right upper columns of the table; relevant correlation marked by bold numbers; r = Pearson coefficient of correlation; N = number of correlated measurements.

In addition there were two patients (patient3; patient4) with apparent discrepancy between FMISO uptake and pO_2_-polarography. Re-evaluation of these patients revealed small, mostly necrotic lymph node metastases with only a small amount of vital tumor tissue and in one case with margining high perfused vessels. After exclusion of these outliers, the absolute value of the corresponding Pearson correlation coefficients increased clearly as shown in Table1 in the right upper columns.

**Figure 2:**
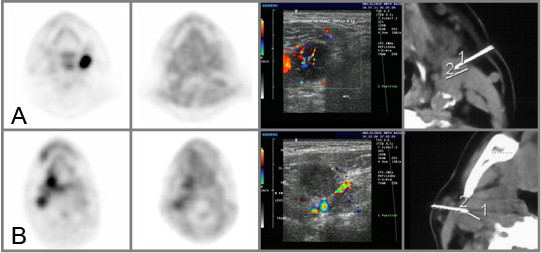
From left to right: transaxial FDG PET-, transaxial FMISO PET-, CDS- and transaxial CT scans in two different patients. A:with a normoxic tumor (pO_2 _≤ 2.5 mmHg = 13.0%, pO_2 _≤ 5.0 mmHg = 15.1%, pO_2 _≤ 10.0 mmHg = 17.0%, median pO_2 _= 36.2 mmHg, mean pO_2 _= 41.1 mmHg; CPD = 12.05%; FDG_SUVmax _= 17.84, FDG_SUVmean _= 14.80; FMISO_T/M _= 1.31, FMISO_T/B _= 1.07). B:with a hypoxic tumorB (pO_2 _≤ 2.5 mmHg = 37.7%, pO_2 _≤ 5.0 mmHg = 48.8%, pO_2 _≤ 10.0 mmHg = 58.1%, median pO_2 _= 5.5 mmHg, mean pO_2 _= 17.7 mmHg; CPD = 2.71%; FDG_SUVmax _= 9.34, FDG_SUVmean _= 8.00; FMISO_T/M _= 1.60, FMISO_T/B _= 0.88). 1/2 = distance measurements for the guidance of the polarographic needle electrode

There was no relevant association between mean or maximum FDG uptake and the different polarographic- as well as the CDS parameters with an absolute value of the Pearson correlation coefficient ranging between 0.056 and 0.241. There was only a moderate association between FMISO_T/B _and FDG_SUVmax _with a Pearson correlation coefficient of 0.403. No relevant correlation between FMISO_SUVmax/mean _and CPD/TP or the polarograhpic parameters could be detected. Figure 2 shows the corresponding results of pO_2 _polarography, CDS, FDG and FMISO PET in a normoxic and a hypoxic tumor.

## Discussion

Results of preclinical and clinical investigations during the last ten years have confirmed that tumor hypoxia precedes malignant progression by several mechanisms, including an increased expression of transcription factors and gene products involved in tumor progression and induction of genomic instability. In those investigations, the transcriptional factor HIF-1 has emerged as a major regulator of adaptive processes (including angiogenesis) that can support tumor cell survival, proliferation, invasion, and tumor spread. It has also been shown that hypoxia can enhance malignant progression and increase aggressiveness through clonal selection [31]. Numerous different approaches have been made in order to identify hypoxia in tumors for clinical use [13].

One standard method for quantifying hypoxia, although it was partly controversially discussed in literature, has been electrode measurement of tumor oxygen tension [4,24,28,29]. Several studies have shown that lower oxygenated tumors are more radio resistant [8,11]. This was recently shown in an international multi-centre study of 397 patients with squamous cell carcinomas of the head and neck [22], identifying pre-treatment tumor hypoxia as an indicator for poor overall survival after radiotherapy. Although it represents a mixture of intra- and intercellular pO_2 _of 30 to 50 cells in front of the probe and not a direct measurement of intracellular oxygenation, it enables an estimation of radio biologically relevant intracellular oxygenation. This could be proved by the use of comet assay and pO_2 _polarograph in anaplastic R3327-AT Dunning prostate tumors, resulting in high correlations between mean pO_2 _and mean comet moment as a parameter of overall strand break induction [26]. Applying pO_2 _histography, an evaluation of intratumoral oxygenation heterogeneity, but not of spatial information is possible. As an invasive method it is confined to superficial, well accessible tumors or lymph node metastases. Evaluating the different methods for determination of tumor oxygenation, we found only slight to moderate correlations between pO_2 _histography and most of the non-invasive measurable parameters. When analyzing the differences in correlation through an evaluation of the scatter plots (Figure 1), we were able to identify four outliers representing the limitations of the different methods.

Comparing the parameters of pO_2 _polarography and CPD there were two patients (patient 1 and 2) with low mean and median pO_2 _values based on inhomogeneous distribution of the different hypoxic fractions. There was a high percentage of readings ≤ 10 mmHg and a low percentage of readings ≤ 2.5 or ≤ 5.0 mmHg. CPD or TP only deliver a mean value of tumor vascularisation or perfusion. The method is therefore unable to reproduce any heterogeneity of tumor oxygenation resulting in a decreased correlation between CPD or TP and the hypoxic fractions ≤ 2.5 and ≤ 5.0 mmHg.

**Figure 3:**
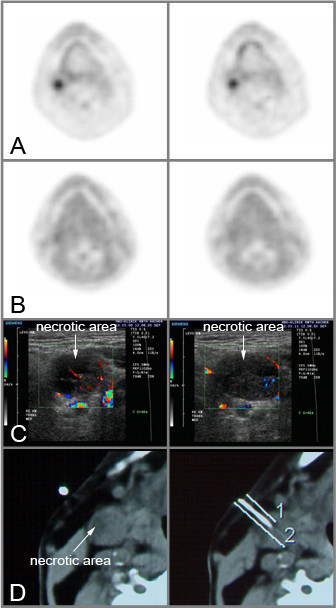
Outlier patient 4, transaxial FDG PET- (A), transaxial FMISO PET- (B), CDS- (C) and transaxial CT (D) scans (pO_2 _= 2.5 mmHg= 43.0%, pO_2 _= 5.0 mmHg = 53.0%, pO_2 _= 10.0 mmHg = 71.5%, median pO_2 _= 3.7 mmHg, mean pO_2 _= 8.8 mmHg; CPD = 2.96%; FDG_SUVmax _= 4.80, FDG_SUVmean _= 4.55; FMISO_T/M _= 1.19, FMISO_T/B _= 1.11). 1/2 = distance measurements for the guidance of the polarographic needle electrode.

As an endpoint for reporting FMISO PET data, we used the ratio between radioactivity in the tumor and reference tissue, consisting of muscle tissue and blood as suggested by Chapman et al. and Kubota et al. [1,16]. It could be shown that the tumor uptake of FMISO was constant between 30 min and 2 h and that the tumor to blood and tumor to muscle FMISO uptake ratios were stable 2–4 h after injection suggesting some retention mechanisms of FMISO within the tumor but not within any normal tissue [16]. Analyzing patient 3, this patient revealed a mostly necrotic lymph node metastasis with only a small amount of margining vital, hyperperfused tumor tissue. Although we tried to ensure electrode measurements in vital tumor tissue by the use of computed tomography guidance, an overestimation of hypoxic fractions by pO_2 _polarography must be expected. Rejecting central necrotic tumor parts from CDS evaluation, we found good correlation between CDS and FMISO parameters. Patient 4 also showed an apparent discrepancy between FMISO uptake and pO_2_-polarography. This patient had a lymph node metastasis with a necrotic part and a part with vital tumor tissue (Figure 3). In this case, it was possible to realize CT controlled pO_2 _polarography and CDS measurement in the vital tumor part. Because of the small vital tumor volume and due to the limited spatial resolution of PET data, a representative measurement of tumor oxygenation by FMISO was not possible. In contrast to CDS parameters with disadvantages in representation of low hypoxic fractions, there were moderate to good correlations between the different hypoxic fractions and FMISO_T/M_. But a decrease in the degree of linear association between FMISO_T/M _and mean as well as median pO_2 _could be detected. Using ^3^H-MISO and a monolayer cell sandwich system with V79 and 9 L cell lines, Hlatky et al. could detect a systematic decrease in the standard deviation of grains per cell when they examined populations of cells further away from the nutrient and oxygen source [9]. An accumulation of MISO below 10.0 mmHg and an inverse relationship of MISO uptake to decreased polarographically measured pO_2 _could be expected. But even this accumulation of MISO below 10 mmHg may explain the lower correlation between FMISO_T/M _or FMISO_T/B _after 2 h and mean or median pO_2_, because of high pO_2 _values leading to higher mean or median pO_2_, which cannot be visualized by FMISO PET. This fact was confirmed by different parameters of pO_2 _polarography showing only slight to moderate correlations between the fraction of pO_2 _values ≤ 2.5 mmHg and the mean or median pO_2_.

The hypothesis that tumor hypoxia has an effect on glucose metabolism is mainly based on experimental data using human cancer cell lines and clinical results and is discussed controversially [2,15,21,34]. Although hypoxia-inducible factor 1 alpha (HIF-1α) is a major regulator of tumor cell adaptation to hypoxic stress, increasing the expression of glycolytic enzymes and proteins [31,33], the FDG uptake was not predictive for tumor hypoxia as assessed by pO_2_-polarography in our series of patients.

Examining blood flow-metabolic relationships, the results analyzing perfusion parameters and glucose metabolism are contradictory [17,20]. According to the progress in tumor molecular genetics in the last decade, biological rational for association of these factors are given by p53 oncogene. The p53 oncogene is known to promote tumor angiogenesis and glucose metabolism. Tumor cells use the tumor suppressor p53, which is usually modified by mutations to debilitate cell cycle controls, to activate hexokinase gene transcription in particular the Type II isoform. This induces the capacity of tumor cells, at least in part, to catabolise glucose at high rates [18,19]. Mutant p53 might also stimulate tumor angiogenesis, indirectly by augmenting the tumor cell proliferation and directly, by up regulating angiogenic factors and down regulating angiogenic inhibitors in the same way [32]. In addition a significant association of intratumoral micro vessel density and p53 protein over expression was described in head-and-neck carcinoma [7]. However, we were not able to show an association between the vascularisation or tumor perfusion seen in CDS images and glucose metabolism as shown by FDG PET parameters, reflecting the problem of adverse effects such as flow based supply of oxygen and glucose, p53 gene based stimulation of tumor angiogenesis and glucose metabolism as well as hypoxia induced HIF-1α expression of glucose transport proteins and hexokinase. It reflects that tumor hypoxia is caused by innumerable, multifactorial, partly contradictory interacting causes and effects complicating detection of therapy relevant hypoxia by the use of clinical examinations. Nevertheless those examinations may enable the transfer of simplified information from cellular micro cosmos into clinical practice.

## Conclusion

Applying CDS for the evaluation of two factors mainly influencing relevant tumor hypoxia as vascularisation (CPD) and perfusion (TP), an estimation of average tissue oxygenation without quantification of oxygenation heterogeneity was possible. The later could be realized by the FMISO parameters especially by FMISO_T/M _after 2 h. It facilitated spatial information, but showed its limits in small tumor lesions caused by spatial resolution of the PET scanner. Although FDG uptake may increase under hypoxic conditions, it could not reliably differentiate hypoxic from normoxic tumors.

Clinical relevance of polarographic determination of tumor oxygenation was elucidated in several clinical studies. But recently the emphasis has been on the potential use of non-invasive approaches. CDS and FMISO PET for estimation of therapy relevant tumor hypoxia represent different non invasive approaches for analysis of tumor hypoxia. But each of these approaches is methodologically limited. Consequently clinical potential must be substantiated in a prospective study, including uniform treatment modalities in order to be more than only bricks in the puzzle of therapy relevant tumor hypoxia.

## Competing interests

Financial competing interests

The pharmaceutical company Hoffmann- La Roche partially financed the costs for FMISO, a substance used to carry out PET scans.

The authors declare that there are no other competing interests.

## Authors' contributions

BG has made substantial contributions to conception and design, acquisition of data, analysis and interpretation of data; MP has been involved in acquisition of data; MP has been involved in acquisition of data; PR has made substantial contributions to conception and design, acquisition of data, analysis and interpretation of data; MZ has made substantial contributions to conception and design, acquisition of data; HJK has been involved in acquisition of data, analysis and interpretation of data; SS has been involved in statistical analysis and interpretation of data; BA has been involved in acquisition of data; CD has made substantial contributions to conception and design; KH has made contributions to conception and design, GMP production of tracer; HHC has made contributions to conception and design, GMP production of tracer; TS acquisition of data, analysis and interpretation of data; PM acquisition of data, analysis and interpretation of data; ED acquisition of data, analysis and interpretation of data; MJE has been involved in analysis and interpretation of data and has made contributions to conception and design.

All authors read and approved the final manuscript.

## Pre-publication history

The pre-publication history for this paper can be accessed here:


